# Efficiency and safety of optic canal unroofing in tuberculum sellae meningiomas: a meta-analysis and systematic review

**DOI:** 10.1007/s10143-023-02151-9

**Published:** 2023-09-12

**Authors:** Peng-Wei Lin, Wei You, Ai-Shun Guo, Zhen-Rong Lin, Yu-Zhe Wang

**Affiliations:** 1 The School of Clinical Medicine, Fujian Medical University, Zhangzhou Affiliated Hospital of Fujian Medical University, Fuzhou, 350122 Fujian Province China; 2 Department of Neurosurgery, Zhangzhou Municipal Hospital of Fujian Province and Zhangzhou Affiliated Hospital of Fujian Medical University, Zhangzhou, 363000 Fujian Province China

**Keywords:** Optic canal unroofing, Tuberculum sellae meningioma, Meta-analysis, Gross total resection, Visual improvement, Complications

## Abstract

Optic canal unroofing (OCU) has gradually become a routine technique for tuberculum sellae meningiomas (TSMs) resection. This meta-analysis aimed to evaluate the efficacy and safety of OCU. A systematic review and meta-analysis of the published literature on this topic from 2003 to 2023 were conducted in accordance with the PRISMA guidelines. Rigorous statistical analysis with a *p*-value was performed for related change in visual improvement, gross total resection (GTR), visual deterioration, and olfactory nerve damage. The study included 15 articles with 384 patients in whom OCU was performed by the transcranial approach (TCA) or the endoscopic endonasal approach (EEA). Of these, 341 patients had preoperative visual loss, and 266 patients had postoperative visual recovery. The overall rate of visual improvement was 0.803 (95% CI: 0.733–0.874, *p* < 0.01). The rate of visual improvement in the EEA and TCA groups was 0.884 (95% CI: 0.803–0.965, *p* < 0.01) and 0.788 (95% CI: 0.700–0.875, *p* < 0.01). Further analysis of classification shows that the rate of visual improvement in Type I: < 2 cm was 0.889(95% CI: 0.739–0.969), Type II:2-4 cm was 0.844(95% CI: 0.755–0.910), Type III: > 4 cm was 0.500(95% CI: 0.068–0.932) and the total was 0.853(95% CI: 0.779–0.927 *p* < 0.01) with low heterogeneity of I^2^ = 20.80%.Twelve studies separately reported GTR with OCU was 293; the rate of GTR was 0.911 (95% CI: 0.848–0.961, *p* < 0.01). And the rate of GTR in Type I: < 2 cm was 0.933(95% CI: 0.817–0.986), Type II:2-4 cm was 0.880(95% CI: 0.800–0.936), Type III: > 4 cm was 0.600(95% CI: 0.147–0.947). The total was 0.897(95% CI: 0.830–0.965 *p* < 0.01) with low heterogeneity of I^2^ = 34.57%. The related complications of OCU were visual deterioration and olfactory nerve damage. Visual decline was reported in nine studies, and the rate was 0.077 (95% CI: 0.041–0.113, *p* < 0.01). Six studies reported olfactory nerve damage, and the overall rate was 0.054 (95% CI: 0.019–0.090, *p* < 0.01). OCU could significantly recover preoperative impaired vision and make GTR easier to achieve, which was also a safe and effective technique in TSM.

**Supplementary Information:**

The online version contains supplementary material available at 10.1007/s10143-023-02151-9.

## Introduction

Tuberculum sellae meningioma (TSM) originates from the tuberculum sellae (TS). Because of its unique anatomical location, TSM can easily invade the optic canal (OC). According to some researchers, OC invasion occurs in 55.1–100% of patients, and a significant number of patients show visual deficits as the primary clinical symptom of TSMs [1–4]. Therefore, the primary treatment goal for TSMs is visual improvement and gross total resection (GTR).

Since Y. Fukado et al. first proposed the concept of decompressing the OC in patients with inflammation and traumatic compression in 1963 [5], several studies have reported its usage for relieving the optic nerve (ON) compressed by the tumor for better visual improvement. However, some surgeons believe that the same visual improvement can be achieved without optic canal unroofing (OCU). Hence, they suggested that performing OCU during surgical resection of the tumor was unnecessary [6]. Given the limited data in the efficacy and safety of OCU in TSM, we conducted a meta-analysis and systematically reviewed related publications in the past 20 years to summarize the impact and safety of OCU in TSM. The meta-analysis included selective published data to provide a rationale for the application of unroofing the OC in appropriate patients.

## Methods

We conducted a systematic review of studies that involved human participants and were published in English in accordance with the Preferred Reporting Items for Systematic Reviews and Meta-Analyses (PRISMA) statement.

### Search strategy

A primary literature search of the PubMed and EMBASE databases was performed for the available observational studies and randomized clinical trials (RCTs) from 2003 to 2023. The search strategy was performed using the following medical subject headings (MeSH) and keywords: “tuberculum sellae,” “suprasellar,” “anterior skull base,” “meningioma,” “TSM,” “optic canal unroofing,” and “decompress optic canal.” Boolean operators (“AND” or “OR”) were used for combination with these terms. If required, the references of the included articles were reviewed to find additional relevant articles. The final results of all available combinations were downloaded into an EndNote library.

### Inclusion and exclusion criteria

All included studies met the following eligibility criteria: (1) all published literature contained original data; (2) the tumor originated specifically from the TS; (3) the surgery involved the procedure of unroofing or opening the OC during resection of TSMs with transnasal or craniotomy approach; (4) the clinical outcome of OCU was described separately; and (5) the sample was clear, and the number of patients in each study was at least 5 for adequately providing the original data.

The exclusion criteria were as follows: (1) case reports, articles describing surgical technology, and academic reports; (2) studies reporting tumors that originated from other anatomical locations: the anterior clinoid process, spheno-orbital, etc.; (3) irrelevant research reports; and (4) ambiguous clinical outcomes or groups.

### Article selection and data extraction

**Fig. 1:**
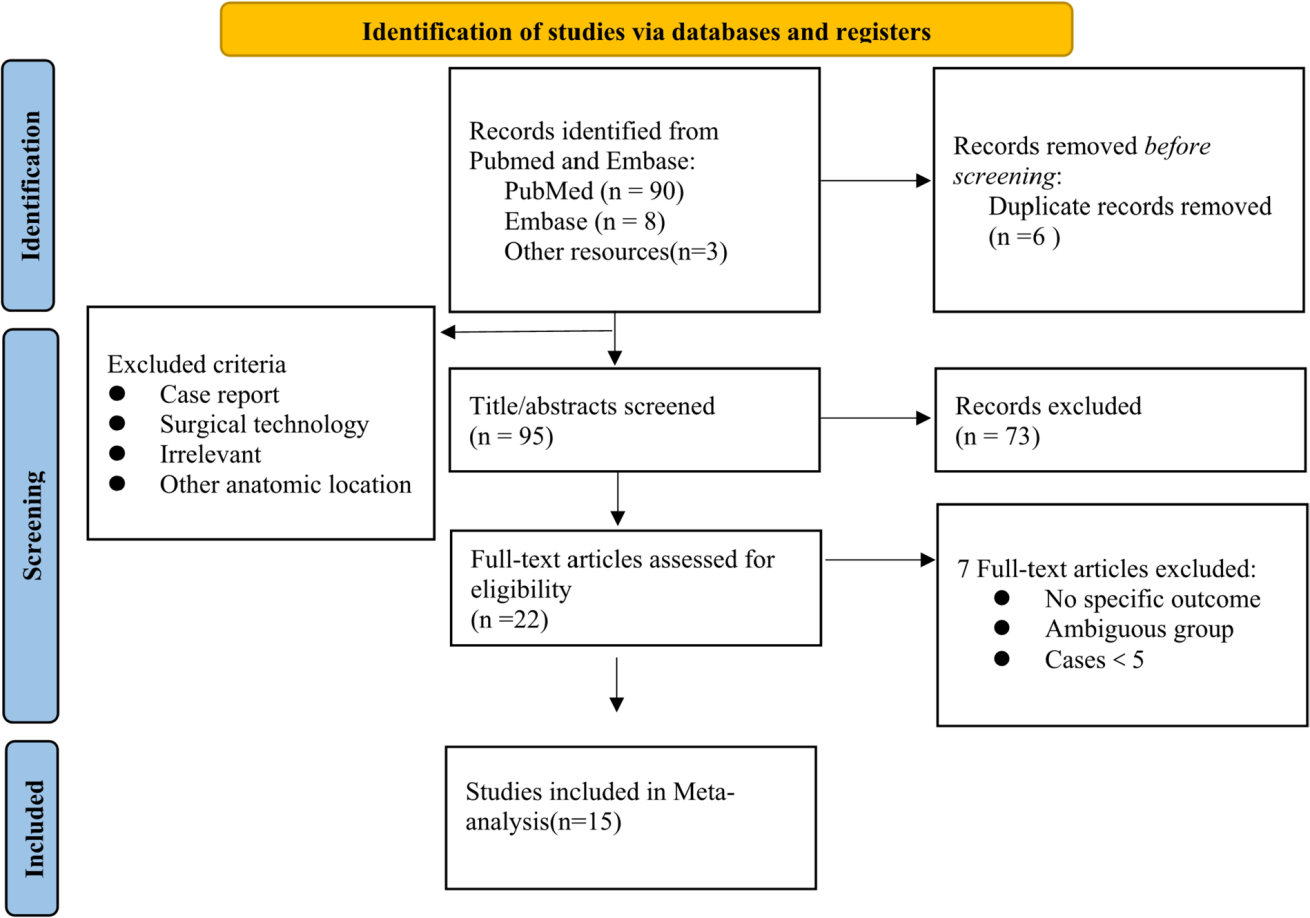
Flowchart

Two authors independently reviewed all search results of articles that met the inclusion criteria. The first phase involved screening relevant titles and abstracts that met the eligibility criteria. Any disagreement was resolved by discussion and through consensus with a third reviewer. In the subsequent stage, full-text reading was performed to identify and extract data based on the inclusion criteria. The included or excluded reasons for the searched articles are shown in Fig. 1.

The data of each selected study that met the inclusion criteria were extracted into a pre-designed table: country, study design, number of patients, age, percentage of females, mean follow-up duration, mean size or volume of tumor, surgical type (transcranial approach [TCA] or endoscopic endonasal approach [EEA]), percentage of preoperative visual loss and unroofing the OC, number of postoperative visual outcome (improved, stable, or worse), and number of patients with GTR and complications.

### Meta-analysis and quality assessment

We used SPSS (version 17.0) to analyze the statistical data. “Metaprop” command, a new program in Stata to perform meta-analyses of binomial data to supplement the metan command, allows computation of 95% confidence intervals (CIs) by using the precise binomial method and the statistical score. The Freeman-Tukey double arcsine transformation of proportions is combined in metaprop [7]. Therefore, the application of metaprop was not affected by the distribution characteristics of the data. Based on this theory, a meta-analysis of the calculated pooled proportion of individual outcomes for the EEA and TCA groups was separately performed. The metaprop command was applied to calculate the corresponding effect size (ES) and stander error of effect size (seES); the outcome was considered statistically significant at the probability value of *p* < 0.05. The results were reported as z value and 95% CIs. Higgins I-squared (I^2^) values were used to assess the heterogeneity of the included studies; studies showing values of ≤ 50% had low or moderate heterogeneity, while those with values of ≥ 75% showed high heterogeneity. Cochrane guidelines for systematic review and meta-analysis and the Newcastle–Ottawa Scale (NOS) were used to assess the quality of the included studies (a score of ≥ 6 was considered high quality).

## Results

### Literature search

**Fig. 2:**
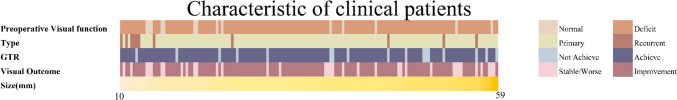
Heatmap showing the characteristic of clinical patients according to tumor size

A total of 101 articles were identified using the abovementioned search strategy. After removing duplicate studies (*n* = 6), the inclusion and exclusion criteria were strictly followed for screening the remaining articles (*n* = 95). Finally, 15 articles [2, 4, 8–20] were eligible for the meta-analysis, which included 5 articles describing the EEA approach [16–20] and 10 articles describing the TCA approach [2, 4, 8–15]. All 15 articles reported retrospective observational human clinical research and were published in English; of these, 14 articles were considered high quality. There were 384 patients with OCU in total, including 314 patients with OCU in the TCA group and 70 patients with OCU in the EEA group. Because the clinical outcomes were not separately analyzed in each article, we did not include all instances in the meta-analysis. The extracted clinical data were used for the meta-analysis. Furthermore, we collected the original clinical data from 8 articles [2, 8, 10, 11, 14, 15, 18, 19] for further subgroup analysis to clarify whether OCU is beneficial for smaller tumors according to the classification of TSMs proposed by Yasargil et al. and Mortazavi et al. [11]. A total of 150 patients' clinical information including: preoperative visual function and types, GTR, visual outcomes, tumor size. We classified them into 3 groups, including Type I: < 2 cm was 45 patients, Type II:2-4 cm was 100 patients and Type III: > 4 cm was 5 patients. And the data characteristics are summarized in Table 1 and Fig. 2

**Table 1:** Study characteristics of tuberculum sellae meningiomas (TSMs) studies

Author	Year	Total cases	Mean age	Meningioma			Approach	OCU	GTR	Visual Outcome		Mean Follow-up (month)	Olfactory nerve damage	NOS
				Mean Size (mm)	OC invasion	Visual loss				Improved	Worsened			
I. Chokyu [8]	2011	34	55.7	24.3	/	32(94.1%)	Bilateral subfrontal	34	27*	28/32*	NR	95.8	1	8
Nozaki [2]	2008	Early:9	46.8	27	6(66.7%)	7(87.5%)	Frontotemporal or Subfrontal	9	7*	7/7*	1*	/	/	6
		Late:7	55	21	7(100%)	5(71.4%)		7	5*	1/5*	3*			
		None:6	59.7	26	4(66.7%)	6(100%)		0	3	2	2			
Chen L [9]	2022	87	49.8	41	73(83.9%)	87(100%)	TCA	87	81*	51/87*	12*	17.4	/	8
Mahmoud [10]	2010	58	56	29	39(67%)	47(81%)	Supraorbital or bifrontal	36	34*	23/29*	2*	NR	3	7
Mortazavi [11]	2016	27	55.4	28.84	15(55.5%)	21(77%)	Frontotemporal	25	25*	19/21*	2*	17.1	4	8
Liu H-C [12]	2015	21	/	/	21(100%)	21(100%)	Subfrontal or Subfrontal temporal	21	NR	13/21*	1*	14.9	/	6
Voznyak [13]	2020	26	49	38	9(52.9%)	17(65.4%)	Contralateral	3	2*	2/3*	1	3–108	/	7
				20	9(100%)	9(34.6%)	Ipsilateral	9	9*	9/9*	/	/	/	
Sade [4]	2009	30	/	23	24(77%)	26(83.9%)	Pterional	30	26*	18/26*	1*	33	/	5
Mathiesen [14]	2006	29	58.3	/	/	24(82.8%)	Frontopterional	29	26*	22/24*	/	/	1	7
Jang W-Y [15]	2012	24	49.5	20.6	/	21(87.5%)	Contralateral	24	24*	17/21*	1*	20.8	2	6
Elshazly K [16]	2018	25	53.9	5.3cm^3^	17(68%)	17(81%)	EEA	25	19*	15/17*	4*	21	/	6
Mallari RJ [17]	2021	20	51	21	12(60%)	17(85%)	EEA	15	18	9/12*	/	45	/	8
Sakata K [18]	2022	6	70	8.4	6(100%)	6(100%)	EEA	6	5*	4/6*	1*	39.3	/	6
Attia M [19]	2012	8	55	27	5(62.5%)	4(50.0%)	EEA	4	3*	3/4*	/	20.6	1	6
Maria K [20]	2014	75	57.3	23	20(26.7%)	61(81.3%)	EEA	20	57	16/17*	2	/	/	8

*OC* optic canal, *GTR* gross total resection, *NOS* Newcastle-Ottawa Scale, *TCA* transcranial approach, *EEA* endonasal endoscopic approach

^*^Dependently described optic canal unroofing

### Visual improvement

We analyzed 15 articles that reported visual improvement in patients with preoperative visual loss to evaluate the effectiveness of unroofing the OC. Ten articles reported 285 patients with preoperative visual deficits in the TCA group who underwent OCU, and the number of patients who experienced visual improvement was 210. Five articles reported 56 patients with preoperative visual loss in the EEA group, and the number of patients with visual improvement was 47. In total, 341 patients had preoperative visual loss, and 266 patients had postoperative visual recovery. The overall rate of visual improvement (postoperative visual improvement /preoperative visual loss cases *100) with unroofing the OC was 0.803 (95% CI: 0.733–0.874; *p* < 0.01). The overall heterogeneity among the included articles was moderate, with I^2^ = 64.81%. We compared the effectiveness of the different approaches of OCU. We then performed subgroup analysis, which showed that the rate of visual improvement in the EEA group was 0.884 (95% CI: 0.803–0.965, *p* < 0.01) with low heterogeneity of I^2^ = 0%, while the ES of visual improvement in the TCA group was 0.788 (95% CI: 0.700–0.875, *p* < 0.01) with high heterogeneity of I^2^ = 72.21%. A total of 136 patients with preoperative visual loss were included for further analysis based on tumor size. Further analysis of classification shows that the rate of visual improvement in Type I: < 2 cm was 0.889(95% CI: 0.739–0.969), Type II:2-4 cm was 0.844(95% CI: 0.755–0.910), Type III: > 4 cm was 0.500(95% CI: 0.068–0.932) and the total was 0.853(95% CI: 0.779–0.927 *p* < 0.01) with low heterogeneity of I^2^ = 20.80%.

### GTR

**Fig. 3:**
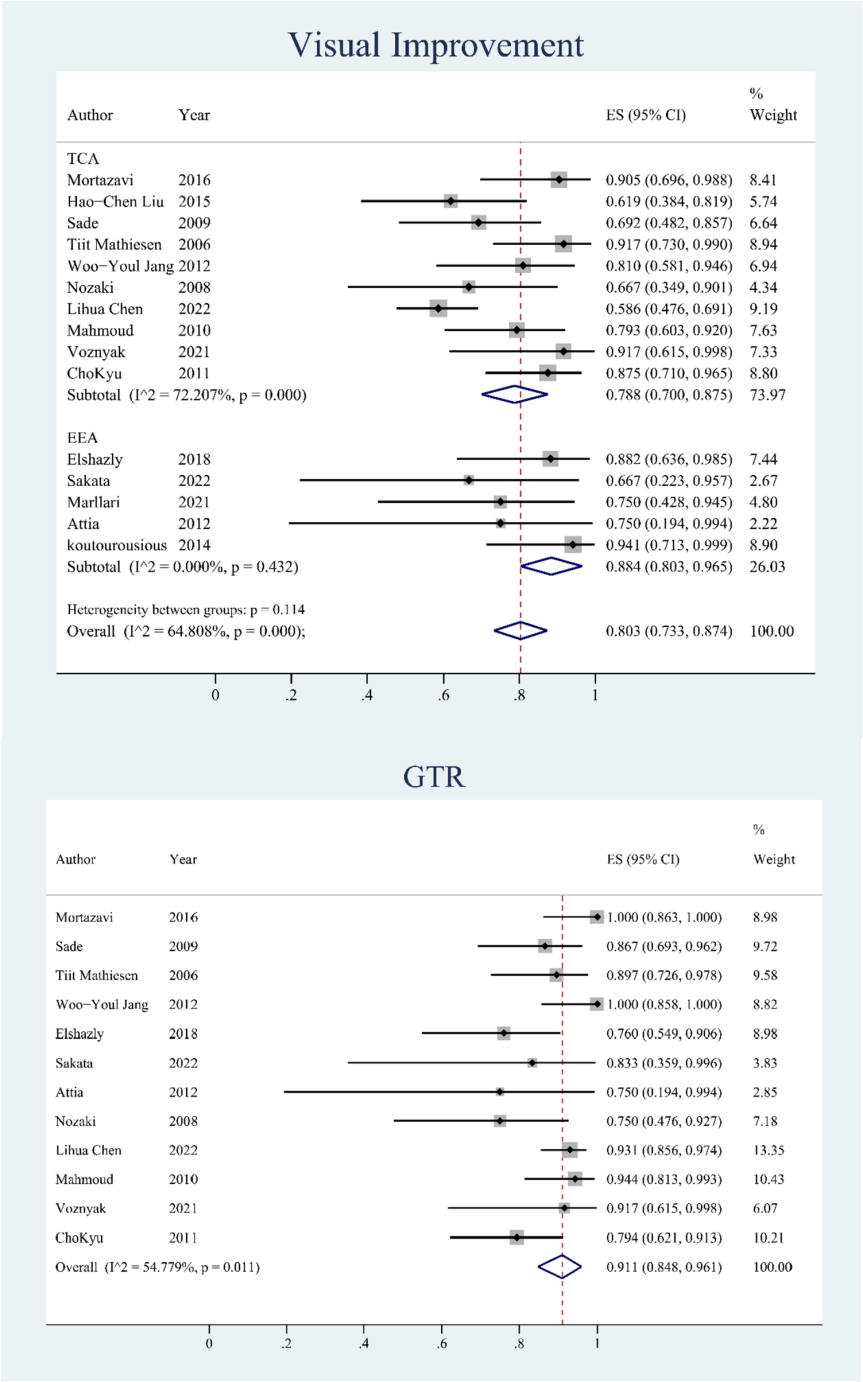
Forest plots showing visual improvement and GTR in optic canal unroofing group. TCA: transcranial approach; EEA: endonasal endoscopic approach; GTR: gross total resection; CI, confidence interval

**Fig.4:**
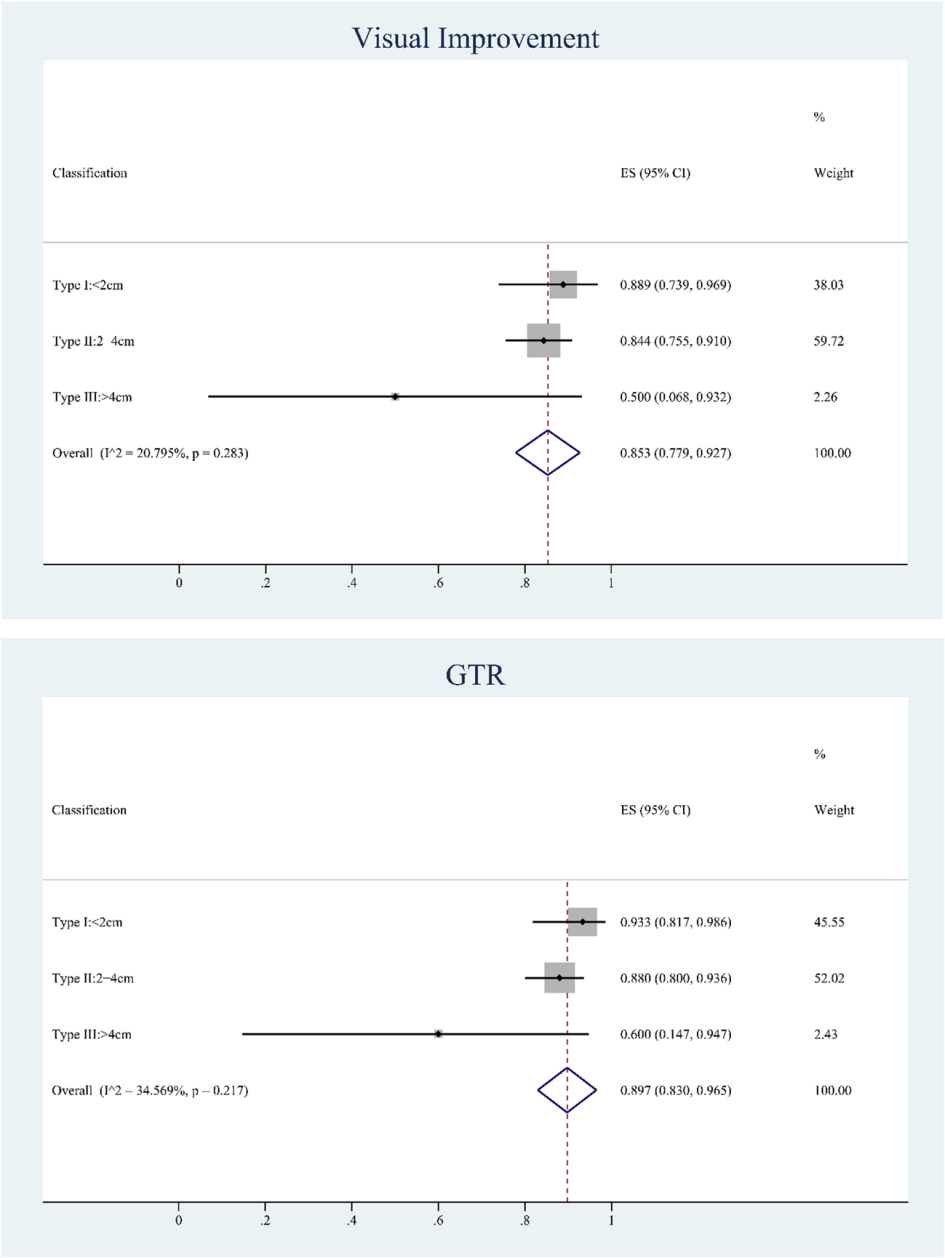
Forest plots showing visual improvement and GTR in optic canal unroofing group according to classification of tumor size

We analyzed 12 studies that separately reported GTR and then calculated the overall number of patients with OCU was 384 and the total number of GTR was 293 [2, 4, 8–11, 13–16, 18, 19]. As shown in Fig. 3, the rate of GTR (GTR cases/total cases*100) was 0.911 (95% CI: 0.848–0.961, *p* < 0.01), and the heterogeneity among the included articles was moderate with an I^2^ = 54.78%. A total of 150 patients with were included for further analysis. Further analysis of classification shows that the rate of GTR in Type I: < 2 cm was 0.933(95% CI: 0.817–0.986), Type II:2-4 cm was 0.880(95% CI: 0.800–0.936), Type III: > 4 cm was 0.600(95% CI: 0.147–0.947) and the total was 0.897(95% CI: 0.830–0.965 *p* < 0.01) with low heterogeneity of I^2^ = 34.57% (Fig. 4).

### Complications

**Fig. 5:**
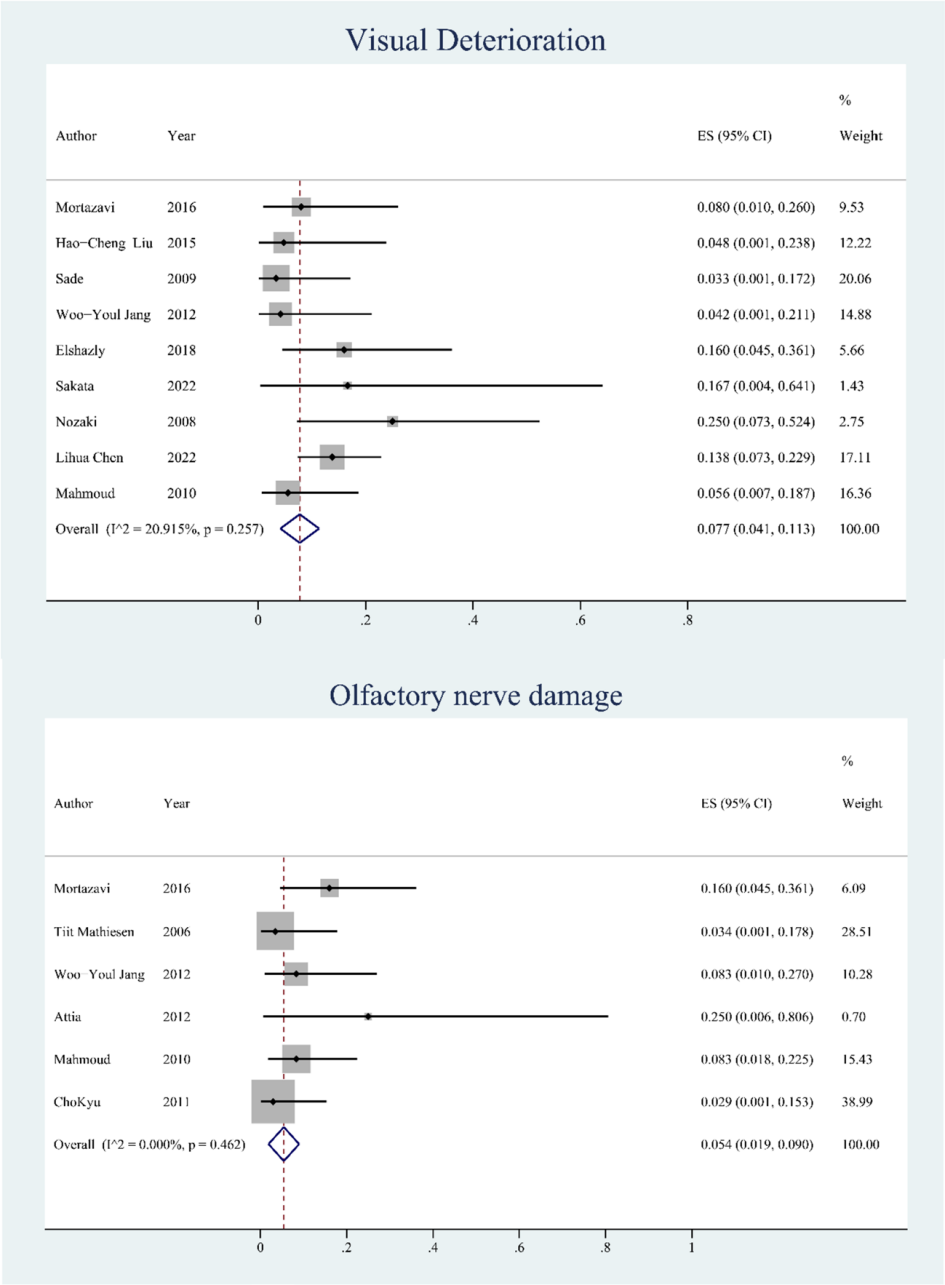
Forest plots showing the postoperative complication of visual deterioration and olfactory nerve damage. CI: confidence interval

The related complications of OCU were mainly visual deterioration and olfactory nerve damage and the incidence rate of complications (events/total cases *100) was calculated. As shown in Fig. 5, in our series visual decline was reported in nine studies [2, 4, 9–12, 15, 16, 18], and the rate was 0.077 (95% CI: 0.041–0.187, *p* < 0.01) with a low heterogeneity (I^2^ = 20.92%). Olfactory nerve damage was reported in 6 studies [8, 10, 11, 14, 15, 19], and the overall rate was 0.054 (95% CI: 0.019–0.090, *p* < 0.01) with a low heterogeneity (I^2^ = 0.000%).

### Publication bias

**Fig. 6:**
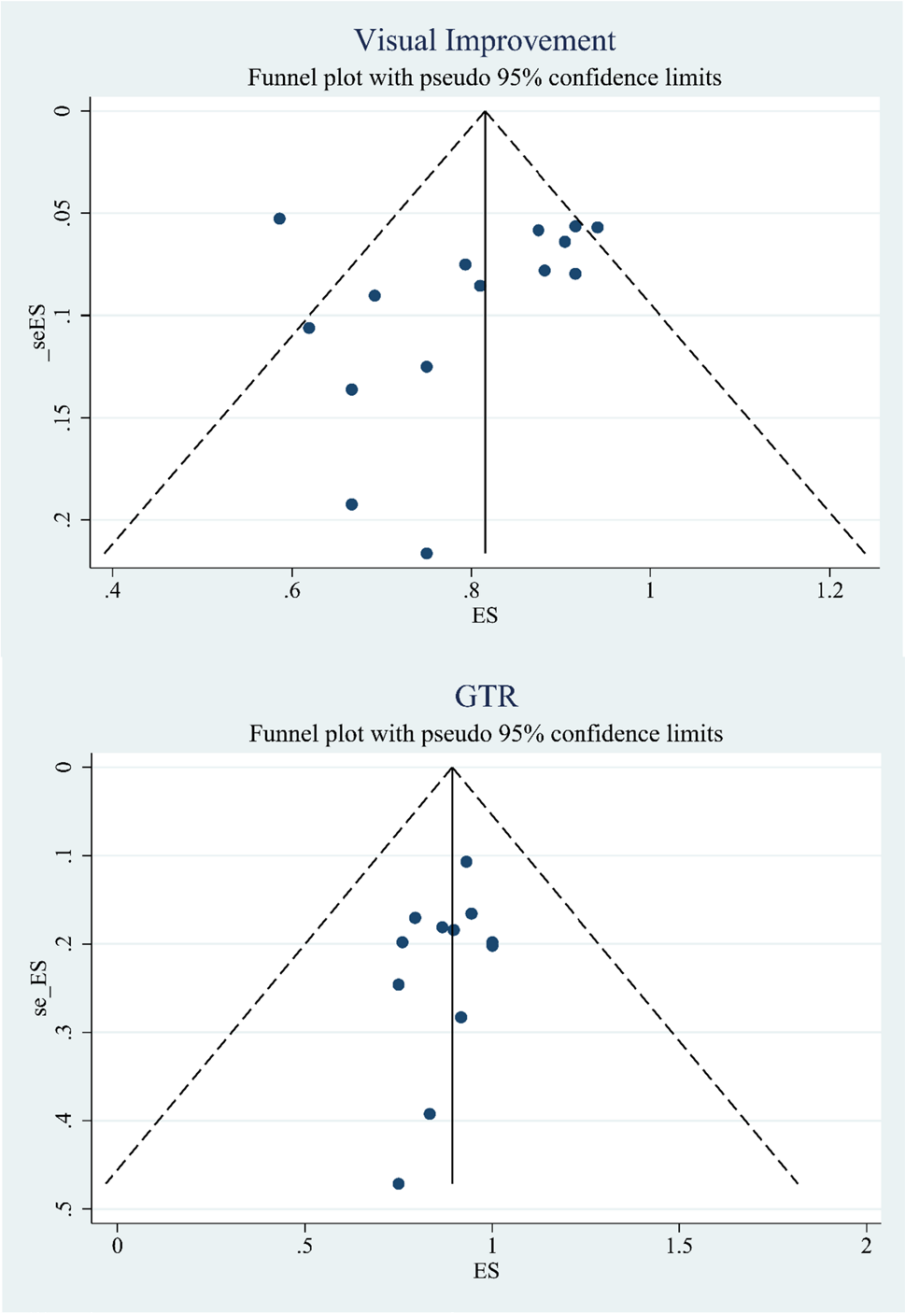
Begg’s funnel plot for publication bias in visual improvement and GTR. Points show the effect size (ES) described in our meta-analysis plotted against the calculated standard error of effect size(seES), dashed line represents 95% confidence interval

**Fig.7:**
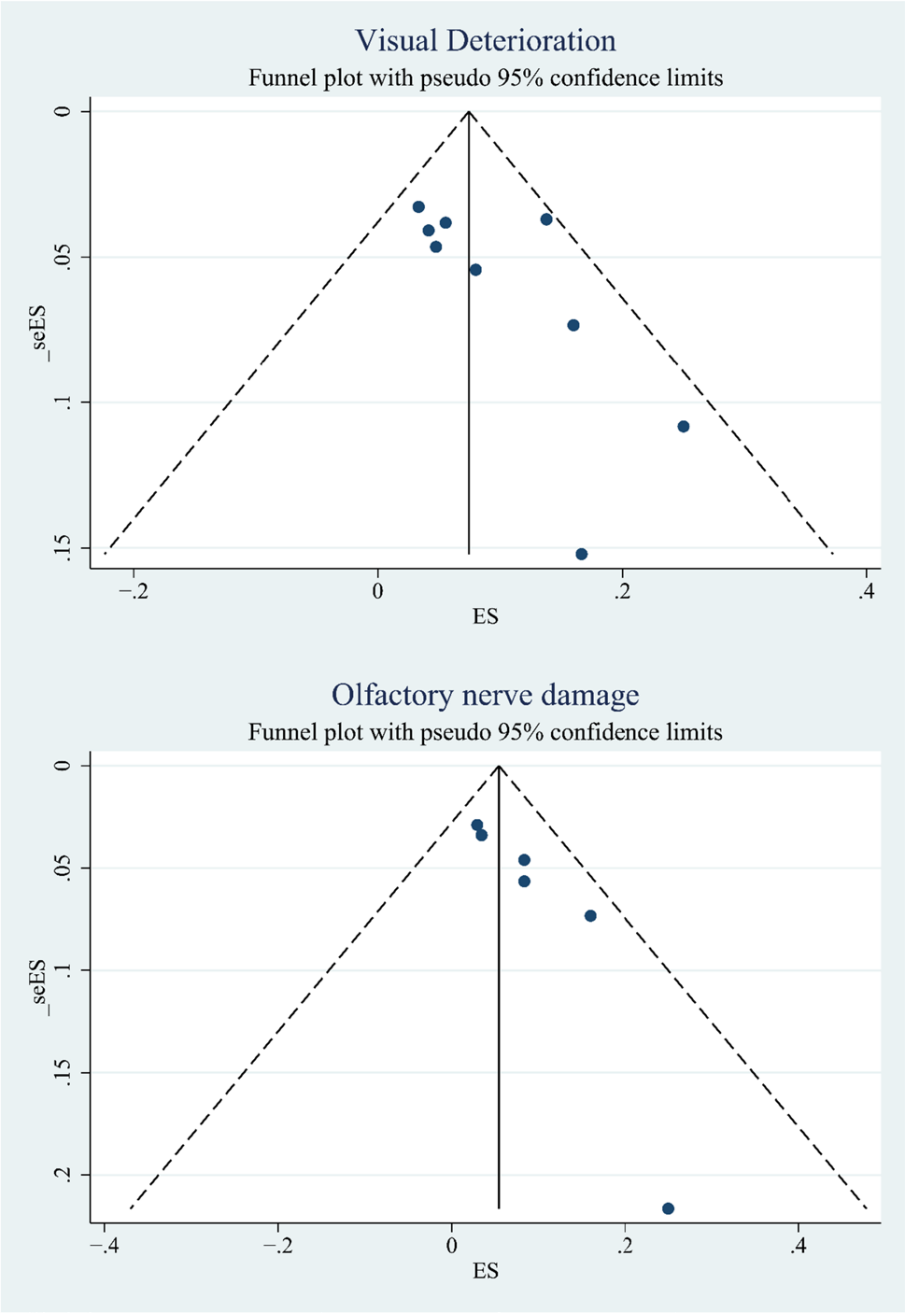
Begg’s funnel plot for publication bias in postoperative complications of visual deterioration and olfactory nerve damage

Begg’s test and Egger’s test were used to analyze the funnel plot to detect the publication bias of the included studies (Table 2). The vertical axis represented the standard error of ES, and the horizontal axis represented the ES (Figs. 6 and 7). The funnel plot of visual improvement was not symmetrical, thus indicating that the limited scattering might be due to publication bias. Egger’s test for visual improvement did not show any publication bias or minor study effects (*p* > 0.05). We suspected that this might be due to the high heterogeneity of the TCA group, which originated from the use of different approaches, including contralateral, supraorbital, and pterional, in the TCA group; consequently, the extent of tumor invasion could not be consolidated. Similarly, GTR, visual deterioration did not show publication bias or small study effects (*p* > 0.05). However, Egger’s test in olfactory nerve damage showed that there were publication bias in this group.

**Table 2:** Results of meta-analysis for overall outcomes

Outcomes	No.of studies	Meta-analysis overall			Egger’s test (*P*-value)
		Effect Size(95%CI)	*P*-value	I^2^	
Visual improvement	TCA:10	0.788(0.700–0.875)	< 0.01	72.21%	0.283
	EEA:5	0.884(0.803–0.965)	< 0.01	0.00%	
	Total:15	0.803(0.733–0.874)	< 0.01	64.81%	
	Type I: < 2 cm	0.889(0.739–0.969)			
	Type II:2-4 cm	0.844(0.755–0.910)			
	Type III: > 4 cm	0.500(0.068–0.932)			
	Total Type	0.853(0.779–0.927)	< 0.01	20.80%	/
GTR	Total:12	0.911(0.848–0.961)	< 0.01	54.78%	0.269
	Type I: < 2 cm	0.933(0.817–0.986)			
	Type II:2-4 cm	0.880(0.800–0.936)			
	Type III: > 4 cm	0.600(0.147–0.947)			
	Total Type	0.897(0.830–0.965)	< 0.01	34.57%	/
Visual deterioration	Total:9	0.077(0.041–0.187)	< 0.01	20.92%	0.972
Olfactory nerve damage	Total:6	0.054(0.019–0.090)	< 0.01	0.00%	0.019

*TCA* transcranial approach, *EEA* endonasal endoscopic approach, *GTR* gross total resection

## Discussion

TSMs tend to affect the bone from where they originated; consequently, regardless of tumor invasion behavior, preoperative CT scanning can routinely detect the hyperosteogeny of the OC leading to ON compression. Many studies have reported different modes of invasion in TSMs. Aria et al. showed that the tumor most commonly tends to inferomedially invade the OC [21]. Koutourousiou et al. reported that the pattern of tumor invasion of the OC in their patient series extended toward the medial aspect direction. A recent study analyzed the characteristics of OC invasion in TSMs [20]. Nimmannitya et al. classified the invasion into four types: type 1: no invasion; type 2: secondary invasion; type 3: partial wall invasion (two subtypes); and type 4: invasion into the superior-medial-inferior walls of the OC. Understanding the patterns of tumor invasion helps develop preoperative surgical strategies and clarifies whether to explore the OC [22].

### The effect of OCU

Our meta-analysis revealed that subtotal visual improvement with OCU in patients with TSMs was 0.803 (95% CI: 0.733–0.874, *p* < 0.01), and the GTR was 0.911 (95% CI: 0.848–0.961, *p* < 0.01), meaning that patients with preoperative visual loss are probably to get visual improvement and GTR after OCU thus confirming the effectiveness of this surgical technique. We confirmed that performing OCU in patients with preoperative visual loss or OC invasion is conductive to visual improvement and the rate of GTR (Table 3).

**Table 3:** Advantages and Disadvantages of different surgical approaches

Surgical approach	Advantages	Disadvantages
Contralateral	1. Direct view of the inferomedial aspect of the affected ON, ICA, and ophthalmic artery without excessive brain retraction 2.Safety of unroofing both OC	1. Difficulty in resection of the tumor extending into the lateral side of ICA 2. Risk of damaging the unaffected ON
Ipsilateral	1. Preserve the unaffected ON and restore the function of affected ON	1. Blind at the inferomedial side of the ON 2. High risk of damaging ON after OCU
Pterional	1. Direct view of the suprasellar region 2. Early decompress affected OC 3. Minimizes damage to the olfactory nerve and anterior cerebral circulation arteries	1. Blind at inferomedial side of the affected OC 2. Potential risk of injury to the branches of ICA
Unilateral subfrontal	1. Direct access to the suprasellar region	1. Blind at the medial aspect of the ipsilateral OC and the inferior aspect of the affected ON
Bilateral subfrontal	1. Direct view of the affected inferior OC and vascular structures	1. Excessive frontal lobe retraction 2. High risk of damage olfactory nerve and CSF leakage 3. Potential risk of postoperative brain edema and venous infarction
Fronto-temporo-orbito-zygomatic	1. Improve the exposure of the skull base minimizing brain retraction 2. Quickly and safely unroofing OC and intradural incision of dural sheath 3. Enable to explore the inferior, medial, and lateral surface of ON	1. Potential risk of necrosis of skin flap, damage of facial nerve or cranial nerve and exophthalmia 2. Transient periorbital edema or diplopia
Endoscopic endonasal	1. Early devascularization and bilaterally unroof the OC 2. Aviod brain retraction 3. Direct visualization of the neurovascular structures of the suprasellar and inferior-medial aspect of ON region	1. High risk of CSF leaks and meningitis 2. Difficulty in resection of large tumor extending beyond lateral ICA and tightly vascular invasion

*ON* optic nerve, *OC* optic canal, *ICA* internal carotid artery, *CSF* cerebrospinal fluid

Furthermore, the clinical outcome was affected by different types of TSMs. The original classification for these tumors was proposed by Cushing and Eisenhardt [23], which classified them into 4 stages. Later, the dimension of the tumor was divided into 3 types by Yasargil. Recently, Mortazavi et al. [11] proposed a precise classification system based on tumor size, optic canal invasion, vascular encasement, brain invasion, previous type and radiation to predict clinical outcomes (Table 4). In addition, Gimmattei et al. applied this classification system to their research and considered it was applicable for predicting visual outcomes [24]. We also attempted to apply this classification system in our meta-analysis. Nevertheless, there is no uniform standard for clinical inclusion criteria. After excluding articles with missing original data, we analyzed only 150 patients from 8 articles and could only divide them into three types based on tumor size. Fortunately, further classification analysis shows that OCU is presumably beneficial for smaller tumors in the rate of visual improvement and GTR. Nevertheless, we could only infer that OCU is probably helpful for smaller tumors because this classification still lacks factors like the OC invasion or vascular encasement, and the clinical outcome depends not only on tumor size. Further researches will focus on the detailed classification of the tumors that allow a suitable comparison between diverse of surgical approaches and predict outcomes or complications [25].

**Table 4:** Proposed Score-Based Classification System for TSM

Parameter	Points			
	0	1	2	4
Size	< 2 cm	2-4 cm	> 4 cm	–
Optic canal invasion	< 5 mm	> 5 mm	Complete	–
Vascular invasion	No	< 180°	> 180°	The maximum for any combination > 4
Brain invasion on MRI	No	Significant	–	–
Previous surgery	No	Yes	–	–
Previous radiation	No	Yes	–	–

However, Park et al. believed that there is no need for additional bone drilling and nerve manipulation around the OC during surgery because of the same clinical outcome of visual improvement and similar tumor resection rate as that achieved for the non-open OC,. And some authors performed OCU only for patients with extensive intracanal extension [6, 26–28].Goldschmidt et al. also reported that only 17% of patients showed visual improvement after OCU [29]. According to recent studies, the involvement of the OC in TSMs facilitates the routine opening of the OC to achieve GTR. We also believe that OCU, early resection of the falciform ligament, and dural dissection of the inner segment of the OC are the primary surgical methods for the decompression of the OC region in TSMs, and these methods can provide more surgical space for the affected ON. If preoperative imaging confirms an OC invasion, it is necessary to open the OC during the surgery to achieve GTR of the intracanal tumor. Even if preoperative imaging shows a negative result for OC invasion but the patient has visual impairment, given the high OC invasion rate, OCU is still recommended to confirm the diagnosis. If the tumor tends to grow and compress the OC, opening the OC for exploration during surgery is also recommended. This view is also supported by other authors. Nozaki et al. proposed that even if preoperative imaging showed no evidence of OC invasion, unroofing of the OC should be considered to explore and decompress the ON and then remove the tumor growing in the canal [2]. Arai et al. emphasized the necessity of OCU for treating preoperative visual loss of patients with TSMs. If there is insufficient surgical space to remove the tumor from the OC, the OC should be opened for decompression, regardless of no apparent evidence of OC invasion [21].

Missed resection of the tumor invading the OC may increase the recurrence rate, which will make it difficult to achieve GTR that is the ideal goal of most surgeons, particularly in TSMs with OC invasion [4, 12, 16]. Tumors are more likely to remain in the OC after surgery without performing OCU, leading to tumor recurrence [9, 14, 30]. By performing OCU to decompress the ON, the tumor in the canal is directly viewed and then resected to protect the integrity of the ON as much as possible. If the OC is invaded by a complex meningioma and the adhesion is too tight to achieve GTR, combining OCU with stereotactic radiotherapy can control tumor recurrence as much as possible [31].

### The timing of OCU

Whether to perform OCU early or late remains a debatable question, which has been discussed in the EANS consensus statement. They summarized that performing OCU before tumor resection seems to be related to excellent visual improvement but depends largely on the severity of OC invasion, the degree of preoperative visual impairment and the surgeon’s technical skills [25]. Nozaki et al. first proposed the importance of the timing of OCU. They reported that the OC should be opened before tumor resection and highlighted that early OCU was one of the single essential factors for postoperative visual improvement [2]. In subsequent studies, some authors performed OCU before tumor resection. They believed that unroofing the OC before tumor resection could decompress the affected ON and restore the blood supply as soon as possible to prevent vision impairment. According to these authors, unified early decompression of the ipsilateral OC is the crucial factor for postoperative visual improvement, which can reduce the tension of the ON and is conducive to postoperative vision recovery. The contralateral OC can also be decompressed if required [11, 14, 20, 32, 33].

In contrast, some authors reported that OCU should be performed only after the removal of the tumor; they considered that adequate decompression of the tumor before opening the OC could avoid damage to the ON. This approach was thought to be not only sufficient but also safe [19, 34, 35]. In some studies that reported postoperative visual deterioration or decline, OC decompression was performed selectively after tumor resection or the OC was not even opened for decompression [35–37]. Lee et al. showed that preoperative ON dysfunction was mainly related to tumor compression of the ON and the surrounding blood vessels, leading to ON compression, ischemia, and even demyelination [38]. Compared to the risk of ON injury caused by OCU, it is essential to decompress the affected ON as soon as possible to relieve the compression and restore the blood supply of the ON. Early resection of the tumor compressing the ON can rapidly relieve the ischemic symptoms and improve impaired vision if a careful operation is performed and direct damage to the ON is avoided [10]. Unfortunately, only a few of the included articles classified the timing of OCU, and the number of articles was insufficient for meta-analysis. However, according to most studies, decompression of the OC before tumor resection was a critical factor for good postoperative visual outcome.

### Different surgical approaches to perform OCU

#### TCA approach

The primary surgical approach for tumor resection depends on the tumor’s anatomical location. Our meta-analysis showed that the overall rate of visual improvement in the TCA group in 10 studies was 0.788 (95% CI: 0.700–0.875, *p* < 0.01). The TCA approach is a traditional surgical resection approach for TSMs, and it provides direct visualization of the tumor and its surrounding structures. As there is sufficient space for manipulation in this approach, it is not limited by tumor size. In the TCA approach, OCU is easy to perform if the tumor extends into the lateral optic apparatus. Substantial visual improvement is relevant to extensive extradural and intradural decompression of the OCs even if the preoperative MRI shows that the tumor has not affected the OC [2, 9]. Dolenc initially pioneered extradural clinoid removal [39], mainly for treating the vascular lesions of the cavernous sinus. Presently, it has become a routine technique that enables the early release of the ON and complete exploration of the OC; hence, this technique with early ON decompression shows good visual improvement [14]. Regarding technical and operational feasibility, some authors advocated TCA approaches such as contralateral, supraorbital, pterional, or supraorbital “keyhole” as well as fronto-temporo-orbito-zygomatic approaches with intradural or extradural ON decompression, including frontotemporal craniotomy, early falciform ligament, resection of the anterior clinoid process, and proximal optic sheath splitting before tumor removal and OC exploration. The skull base technique involving the extradural posterolateral orbitotomy, OCU, and anterior clinoidectomy is a representative and easy-to-perform technique for treating the affected OC, which enables clear visualization of the ON, opticocarotid, and the interoptic and carotid-oculomotor triangles [4, 33, 40]. Table 3 lists the unique advantages and disadvantages of the different surgical approaches.

#### EEA approach

The EEA is a recently developed approach. Several studies supported this approach as a safe surgical indication for TSMs. Compared to the TCA, the EEA can easily enter the tumor directly and avoid brain retraction, which facilitates devascularization and drilling of the OC toward the orbital bilaterally [41]. The EEA is also the most appropriate approach to dissect the undersurface of the affected optic apparatus, which tends to be a blind area when using the TCA. Our meta-analysis reported that subtotal visual improvement in the EEA group in the 5 articles was 0.884 (95% CI: 0.803–0.965, *p* < 0.01).

Rose and Lund first applied endoscopic endonasal decompression of the OC in patients with spheno-orbital meningiomas invading the OC [42]. In subsequent studies, some authors found that the anterior clinoid process and the optic strut surround the OC, because of which the OC entrance lacks sufficient space for displacement; consequently, the ON surrounding the OC entrance is severely compressed and twisted. Moreover, the tumor generally compresses the ON from the inferomedial side to the superolateral side, leading to the ON mostly being squeezed superolateral at the optic chiasm; thus, the release of the ON through the TCA is challenging and complex [43]. Several researchers have agreed that TSMs involving the OC can be resected safely through the EEA by decompressing the ON to 270°, thereby improving visual outcome. EEA eliminates brain retraction, which reduces the risk of postoperative seizures and provides direct visualization of the suprasellar, subchiasmatic perforators, and inferomedial OC region to recognize the neurovascular structures clearly, particularly the branches of the superior hypophyseal artery (SHA); this allows to perform GTR with minimal neurovascular manipulations and reduces the tumor recurrence rate [3, 19, 20]. Recently, Abhinav et al. proved that decompression of the OC and division of the falciform ligament with the extensive endonasal approach was feasible and safe. They described in detail the osseous OC anatomy combined with the transcranial and endonasal endoscopic view. They proposed lateral optic carotid recess (LOCR) as a landmark to identify the pre-foraminal ON and facilitate the preoperative surgical consideration regarding the extent of dural opening and OC decompression [44]. Hence, it is imperative to decompress the OC through the EEA, which provides a direct view and successfully enables unroofing of the OC safely and completely without a blind area, even for TSMs affecting the bilateral OC.

In contrast, a relative number of authors argued that the EEA could not safely and completely resolve TSMs involving the OC [10]. Some authors also highlighted that there was an imperatively increased risk of cerebrospinal fluid (CSF) leakage [10, 19, 45, 46]; moreover, some previous meta-analyses reported that the rate of CSF leakage was 32% [47] and 21% [48]. The disadvantages of the EEA reported in other studies include the difficulty in obtaining distal control, limitations in vascular repair (clip application), and inappropriate resection of tumors originating from the anterior clinoid process (lateral to the ON) or extending along the lateral aspects of the OC [44]. Fortunately, following the development of useful surgical techniques and devices, such as the Hadad-Bassagasteguy flap and gasket seal, the rate of CSF leakage decreased from 22% between 2004 and 2010 to 4% between 2016 and 2020 [49]; this increase promoted the application of the EEA in appropriate patients with TSM. In selective patients, TSMs with OC involvement cannot be considered a limitation for resection in the EEA. Sometimes the EEA performs better than the TCA in terms of less invasive manipulation, shorter hospital stay, and better visual improvement [43, 48]. However, we still cannot conclude whether the EEA or TCA is more suitable for performing OCU in TSMs; this is due to the lack of sufficient clinical data and RCTs that are difficult to conduct because of varying surgical indications.

### Surgical indications of OCU

The EANS consensus concluded that routine OCU not only depends on the adequate imaging to estimate the tumor invasion patterns but also remains to be decided base on tumor extensions and surgeons’ preference [25]. There is still no consensus regarding surgical indications for exploring the OC in TSMs, together with the lack of supporting clinical evidence. OCU is not recommended for all patients with TSMs. Given the limitations of neuroimaging techniques, some subtle TSMs invading the OC cannot be fully evaluated by imaging. We summarized the surgical indications for exploring the OC as follows: (1) preoperative neuroimaging evidence of OC involvement (CT or MRI); (2) visual acuity below 0.1 or visual field deficit regardless of whether preoperative neuroimaging detects OC involvement; (3) peak systolic velocity of the central retinal artery in the orbital color Doppler imaging (CDI) test is ≤ 8 cm/s; and (4) intraoperative exploration shows a tendency of the tumor to invade the OC or ON [12, 50]. These indications must be confirmed by supporting clinical evidence. Future studies in our institute will also aim to verify the appropriate approach and surgical indications for OC opening.

### Safety of OCU

Bone drilling is necessary for OCU, which allows better visualization and possibly maximum resection [9, 51]. However, drilling-related injuries, including direct trauma or overheating injury to the ON or the surrounding structures, lead to the potential risk of damaging the ON, the carotid artery, and the olfactory nerve during OC drilling [14, 15]. We concluded that the related complications of OCU were mainly visual deterioration and olfactory nerve damage. In our series, visual decline was observed in nine studies, and the ES was 0.077 (95% CI: 0.041–0.187, *p* < 0.01). Theoretically, visual deterioration may be caused by mechanical and thermal injury to the ON and interruption of blood supply to the ON during drilling [52, 53]. However, drilling alone does not usually lead to visual deterioration. Tight adhesion and continuous compression of the ON will increase the manipulation of the ON and lead to visual damage. Based on our collected data, our discussion is limited to the low incidence of visual deterioration due to OCU. Fortunately, such visual deterioration is transient and will recover in the subsequent 6–12 months. Some studies have suggested that generous irrigation could be used to prevent thermal injury of the ON when a high-speed drill was used for drilling [14, 35, 50, 53].

Another common complication is olfactory nerve damage. In many cases, unilateral olfactory nerve resection is chosen for extensive OCU [8]. According to our meta-analysis, the ES of olfactory nerve damage was 0.054 (95% CI: 0.019–0.090, *p* < 0.01). Nevertheless, postoperative hyposmia rarely occurs in these patients, thus implying that unilateral olfactory nerve could be reliably preserved to maintain normal olfaction by using advanced microsurgical delicate manipulation [8, 15]. Relatively rare complications such as cranial nerve damage, diabetes insipidus, hemorrhage in the surgical areas, or seizures are difficult to be analyzed and recorded because of their low rates.

## Conclusions

Our meta-analysis showed that OCU could significantly recover preoperative impaired vision and make GTR easier to achieve in selective patients with TSMs. And OCU is also presumably advantageous to smaller tumors, which still needs the proper and validated classifications in tumors to predict clinical outcomes. Exploring the affected OC before tumor resection was beneficial for early decompressing the ON and restoring the blood supply to the compressed ON as soon as possible to prevent vision impairment. Both TCA and EEA could perform OCU, and the appropriate approach should be selected according to the characteristics of TSMs. OCU was a safe and effective technique because the incidence of complications was relatively low. Strict surgical indications can help rationalize the application of OCU and further improve the prognosis of patients.

## Strengths and limitations

Our meta-analysis strictly followed the selection criteria and PRISMA guidelines. Our research could be the first meta-analysis of the efficacy and safety of OCU in TSMs. In addition, this review was registered (ID: CRD42023397984) in PROSPERO (International Prospective Register of Systematic Reviews). However, we could not find any RCTs from large clinical centers for analysis. Most of the included studies were retrospective observational studies, and their data were relatively scattered or no uniform inclusions; therefore, we could only select a single meta-analysis to analyze the clinical data. Understandably, OCU is relatively challenging to perform during tumor resection because of time consumption and is not suitable for all patients. Neurosurgeons use different surgical techniques and standards for selecting a suitable approach. The collection of a large amount of data requires multicenter cooperation for a long time. Additionally, there is no consensus regarding the inclusion criteria and surgical indications for OCU, and clear guidelines for patients with TSMs, such as the timing, indication, and scope of OCU, are lacking. Our present meta-analysis was limited to the currently published articles, which illustrated the efficiency and safety of OCU, with the hope to provide a new idea for resecting TSMs invading the OC and to improve patient prognosis.

### Supplementary Information

Below is the link to the electronic supplementary material.

Supplementary file1 (DOCX 14 KB)

## Data Availability

This study does not include any studies with human or animal participants performed by any of the authors. Data availability is not applicable to this article as no new data were created or analyzed in this study.
