# Supplementary material for: Efficiency and safety of optic canal unroofing in tuberculum sellae meningiomas: a meta-analysis and systematic review
Source: Neurosurg Rev. 2023 Sep 12;46(1):240. doi: 10.1007/s10143-023-02151-9 (PMC10497650; doi:10.1007/s10143-023-02151-9)
Supplement: Supplementary file 1 — Supplementary file1 (DOCX 14 KB) [file 10143_2023_2151_MOESM1_ESM.docx]

The search formula was as follows: “Tuberculum[Title/Abstract] OR sellar[Title/Abstract] OR sella[Title/Abstract] OR sellae[Title/Abstract] OR Planum[Title/Abstract] OR Sphenoid*[Title/Abstract] OR anterior skull base[Title/Abstract]) AND (Meningioma*[Title/Abstract] OR meningioma[MeSH Terms] OR meningeoma*[Title/Abstract] OR meningeal neoplasms[MeSH Terms] OR TSM[Title/Abstract]) AND ((optic canal*[Title/Abstract] OR optic nerve OR optic foramen[Title/Abstract]) AND (decompress*[Title/Abstract] OR drill*[Title/Abstract] OR unroof*[Title/Abstract])) Filters: from 2003 to 2023.”
